# Comparative Single-Nucleus Transcriptomic Analysis of the Intact Left Ventricle in *Acomys cahirinus* and *Mus musculus*

**DOI:** 10.3390/genes17091071

**Published:** 2026-09-04

**Authors:** Nikita Filatov, Kirill Kokorichev, Dmitry Tychinin, Taisiya Voronina, Elena Shagimardanova, Konstantin Dergilev, Pavel Makarevich, Andrey Kiyasov, Vsevolod Tkachuk, Mary Woroncow, Yelena Parfyonova, Airat Bilyalov, Oleg Gusev

**Affiliations:** 1 National Medical Research Centre of Cardiology Named After Academician E.I. Chazov, Ministry of Health of the Russian Federation, Moscow 121552, Russia; ns.filatov@yandex.ru (N.F.); kirillkoko@gmail.com (K.K.);; 2 Institute of Fundamental Medicine and Biology, Kazan Federal University, Kazan 420008, Russia; 3 Institution “Centre for Strategic Planning and Management of Biomedical Health Risks”, Federal Medical Biological Agency, Centre for Strategic Planning of the FMBA of Russia, 10-1 Pogodinskaya Street, Moscow 119121, Russia; 4 Life Improvement by Future Technologies (LIFT) Center, Moscow 121205, Russia; 5 Genomics and Bioimaging Core Facility, Skolkovo Institute of Science and Technology, Moscow 143026, Russia; 6 Center for Regenerative Medicine, Medical Research and Education Institute, Lomonosov Moscow State University, Moscow 119192, Russia; 7 Faculty of Medicine, Medical Research and Education Institute, Lomonosov Moscow State University, Moscow 119192, Russia; 8 Loginov Moscow Clinical Scientific Center, Moscow 111123, Russia; 9 Intractable Disease Research Center, Graduate School of Medicine, Juntendo University, Tokyo 113-8421, Japan

**Keywords:** *A. cahirinus*, single-nucleus RNA sequencing, ventricular cardiomyocytes, interspecies transcriptomic differences

## Abstract

Background/Objectives: *Acomys cahirinus* exhibits more favorable cardiac remodeling after ischemic injury than *Mus musculus*, yet the molecular organization of the intact myocardium remains poorly characterized. This study aimed to identify baseline interspecies differences in the adult left ventricle, with particular emphasis on ventricular cardiomyocyte transcriptional states. Methods: Intact left ventricles from adult male *A. cahirinus* and *M. musculus* were analyzed by single-nucleus RNA sequencing using three biological samples per species. Cross-species integration was followed by cell type annotation, pseudobulk differential expression analysis, Augur-based assessment of interspecies separability, coexpression module analysis, and focused reintegration analysis of ventricular cardiomyocytes. Cardiomyocyte profiles were further compared with published embryonic and early postnatal *M. musculus* heart datasets. Results: Both species contained comparable major myocardial cell populations, whereas marked interspecies transcriptional differences persisted within individual cell types. Ventricular cardiomyocytes exhibited the largest number of differentially expressed genes and were classified into three transcriptional states: contractile, matrix-associated, and alternative. Adult *A. cahirinus* cardiomyocytes retained a mature cardiomyocyte phenotype but showed greater enrichment of developmental, morphogenetic, and Hippo-associated gene programs than adult *M. musculus* cardiomyocytes. Their transcriptional profile did not correspond to embryonic or early postnatal mouse states. The integrated atlas further identified interspecies differences in fibroblast-associated and extracellular matrix-related programs. Conclusions: The intact myocardium of *A. cahirinus* exhibits cell type-specific transcriptional differences relative to *M. musculus*. Ventricular cardiomyocytes display a distinct mature transcriptional configuration rather than a direct immature state, which may contribute to the favorable response of *A. cahirinus* to myocardial injury.

## 1. Introduction

Maintenance of the structural and functional integrity of the myocardium is essential for normal cardiac contractility. However, the capacity for recovery after injury varies substantially among species and remains severely limited in mammals, including humans. In humans and laboratory mice, ischemic injury predominantly results in cardiomyocyte loss, fibrotic scar formation, and subsequent pathological remodeling, which do not restore the original functional organization of the tissue [1,2,3,4]. Animal species in which the tissue response to injury does not result in replacement fibrosis but is accompanied by more effective repair and remodeling provide informative experimental models [5,6,7]. Studies of such models can identify biological features potentially associated with more favorable outcomes after myocardial injury.

Spiny mice of the genus *A. cahirinus* are regarded as a model of regenerative healing in adult mammals and exhibit scar-free skin wound healing and ear pinna regeneration, with restoration of complex tissue structures, including hair follicles, sebaceous glands and cartilage [8,9,10,11,12,13]. After experimentally induced myocardial infarction, *A. cahirinus* have higher survival, smaller scars, greater vascularization, and partial recovery of cardiac function compared with *Mus musculus* [14,15].

Available data on the *A. cahirinus* heart suggest that cardiomyocyte properties may contribute to a more favorable response to ischemic injury. These properties include features of a less mature cellular state and distinct metabolic characteristics [16,17]. The observed phenotype may also involve other myocardial cell populations, including fibroblasts, in addition to cardiomyocytes. In particular, myocardial infarction in *A. cahirinus* is followed by specific features of scar tissue and the extracellular matrix that are associated with less pronounced pathological remodeling and preservation of cardiac function [17,18]. However, baseline molecular programs in the intact *A. cahirinus* heart, including the states of its major cell populations before injury, remain insufficiently characterized.

These findings raise the question of whether the more favorable post-infarction phenotype of *A. cahirinus* is determined exclusively by injury-induced programs or whether some of these features are already present in the intact heart. The myocardium of *A. cahirinus* and *M. musculus* may therefore differ not only in their responses to ischemic injury but also in the baseline molecular organization of intact tissue. To examine this possibility, we performed a comparative single-nucleus transcriptomic analysis of intact left ventricles from *A. cahirinus cahirinus* and *M. musculus*. We hypothesized that interspecies differences in the uninjured heart would be reflected primarily in distinct configurations of cell type-specific transcriptional programs rather than in marked changes in cellular composition, and that these programs could influence the subsequent myocardial response to injury.

## 2. Materials and Methods

### 2.1. Animals and Ethical Approval

The study included intact left ventricles from adult male *A. cahirinus* and *M. musculus*, with three biological samples obtained from each species. The animals were housed under standard animal facility conditions with controlled temperature and lighting and had ad libitum access to food and water. Euthanasia was performed in a sealed induction chamber using isoflurane in oxygen delivered via a calibrated vaporizer. The isoflurane concentration was maintained at ≥5% until loss of response to a noxious stimulus and the onset of apnea, after which anesthetic delivery was continued for at least 60 s. The heart was rapidly excised under aseptic conditions. The left ventricle was dissected on a chilled surface and used for subsequent sample preparation. All animal procedures were approved by the Ethics Committee of the National Medical Research Center of Cardiology named after Academician E.I. Chazov of the Ministry of Health of the Russian Federation (protocol no. LA/20.12.24, approved 20 December 2024).

### 2.2. Single-Nuclei RNA Seq Library Preparation and Sequencing

The dissected left ventricle was rapidly snap-frozen in liquid nitrogen and stored at −80 °C until further use. For nuclei isolation the tissue was placed on prechilled Petri dish and minced in ice-cold lysis buffer (10 mM NaCl, 10 mM MgCl_2_, 10 mM Tris-HCl pH 7.4, 0,01% Nonidet P40 Substitute). Tissue was homogenized using gentleMACS Octo Dissociator and filtered through a 30 μm strainer (MACS SmartStrainers 30 μm, cat. no. 130-098-458; Miltenyi Biotec B.V. & Co. KG, Bergisch Gladbach, Germany) on ice. Collected nuclei suspension was collected and centrifuged at 700× *g* for 5 min at 4 °C. Supernatant were carefully removed and pellet was resuspended twice in 1 mL of 1% bovine serum albumin (BSA) (10 mg/mL BSA in Mg^2+^-free, Ca^2+^-free, RNase-free phosphate-buffered saline (PBS)) with 12.5 μL of RNAse inhibitor (RiboLock RNase Inhibitor (40 U/μL), 2500 U, Thermo Fisher Scientific UAB, Vilnius, Lithuania; cat. no. EO0384). An aliquot of the resulting nuclei suspension was analyzed by SYBR Gold and propidium iodide staining by microscopy using fluorescent microscope Axio Vert.A1 (Carl Zeiss Microscopy GmbH, Jena, Germany). Approximately 12000 nuclei from each sample were loaded onto a Seek One^®^ DD platform (Beijing Seek Gene Biotechnology Co., Ltd., Beijing, China). The SeekOne DD Single Cell 5′ Transcriptome-seq Kit v1.1 (Beijing Seek Gene Biotechnology Co., Ltd., Beijing, China) was used to generate single-cell libraries according to the manufacturer’s instructions. Library quality control was performed using Bioanalyzer 2100 (Agilent Technologies, Santa Clara, CA, USA). The sequencing of libraries was performed on an Illumina Novaseq 6000 (Illumina, Inc., San Diego, CA, USA) platform in a 29 + 150 base paired-end mode.

### 2.3. Preprocessing and Analysis of Single-Nucleus RNA-Seq Data

Raw FASTQ files were processed locally using theRNA module of the SeekSoulTools package (v.1.2.1; SEEKGENE, Beijing, China). For each sample, we performed cell barcode and unique molecular identifier (UMI) recognition, read alignment to the corresponding reference genome, and gene-level expression quantification. For *M. musculus*, we used the GRCm39 reference genome and the corresponding gene transfer format (GTF) annotation. For *A. cahirinus*, we used our own genome assembly with gene annotation that included paralogous genes. The --include-introns parameter was used to account for intronic reads. The output for each sample was a gene-by-cell count matrix, which was then used for quality control and downstream analysis using the scanpy library (v.1.11.5). *A. cahirinus* and *M. musculus* datasets were processed independently. Nuclei with fewer than 200 detected genes, a total UMI count of fewer than 500 or more than 25,000, more than 6000 detected genes, or a proportion of mitochondrial or ribosomal transcripts exceeding 8% were excluded from the analysis. Genes expressed in fewer than 10 cells were removed from the analysis. Potential duplicates were identified using sc.external.pp.scrublet with a threshold of 0.25. After cell filtering, hemoglobin and ribosomal genes were removed from the count matrix. For each species separately, normalization was performed using sc.pp.normalize_total (target_sum = 10,000), followed by a log1p transformation using sc.pp.log1p. Highly variable genes were identified using the sc.pp.highly_variable_genes function (n_top_genes = 3500) for each species separately. For interspecies integration, we used the intersection of highly variable genes (HVGs), which comprised 2144 genes. Interspecies integration of *A. cahirinus* and *M. musculus* data was performed using sysVI (scvi-tools v.1.3.3). The model was trained on a normalized log1p-transformed expression matrix based on the common set of highly variable genes. Species was used as the system covariate, and the sample ID was used as an additional categorical covariate. To ensure reproducibility, scvi.settings.seed was set to 1, and the model was trained with the parameter z_distance_cycle_weight set to 2. The resulting latent representation sysVI was used to construct a k-nearest neighbors (KNN) graph using the sc.pp.neighbors function (n_neighbors = 15), followed by UMAP visualization and Leiden clustering.

### 2.4. Cell Type Annotation

Cell types were annotated manually based on the specific expression of established marker genes and differentially expressed genes characteristic of each Leiden cluster. Cluster-specific marker genes were identified using sc.tl.rank_genes_groups. Clusters with ambiguous annotations or evidence of low quality were excluded based on coexpression of markers from different cell populations, the number of detected genes, and the proportion of mitochondrial transcripts.

### 2.5. Differential Expression Analysis

Differential expression analysis between *A. cahirinus* and *M. musculus* samples was performed at the pseudobulk level separately for each cell type. Raw counts were aggregated by summing UMIs across a combination of cell type, species, and sample. Only pseudobulk profiles containing at least 20 nuclei and cell types with at least two pseudobulk replicates per species were included in the analysis. Prior to differential analysis, genes were filtered based on minimum representation. Genes with a nonzero read count in at least two pseudobulk samples and a total read count of at least 10 were included in the analysis. Differential expression was calculated using PyDESeq2 v.0.5.2 with a ~condition design, where condition corresponded to species. For downstream filtering and visualization of differentially expressed genes, the following thresholds were used: adjusted *p*-value < 0.05, |log2FoldChange| ≥ 1, and gene expression in at least 5% of the nuclei of at least one of the compared species.

### 2.6. Coexpression Gene Module Analysis

Coexpressed gene modules were identified using the Hotspot v.1.1.1 software package. The analysis was performed on an integrated dataset comprising all annotated cardiac cell types. Highly variable genes were used as the initial feature space. Hotspot was used to identify genes with locally autocorrelated expression in the cellular graph and to subsequently group these genes into coexpression modules, taking into account UMI correction based on a negative binomial model.

To assess the local structure of the data, a k-nearest neighbors (KNN) graph was constructed using the function ‘hs.create_knn_graph (n_neighbors = 30, weighted_graph = False)’. The local autocorrelation of gene expression was then calculated using the function ‘hs.compute_autocorrelations’. To identify modules, we selected genes with significant autocorrelation (false discovery rate (FDR) < 0.001), ranked by Z-score. Up to 500 of the most autocorrelated genes were included in the analysis. Coexpression modules were generated using the function ‘hs.create_modules’ (min_gene_threshold = 15, core_only = True, FDR_threshold = 0.05). Module scores at the cellular level were calculated using ‘hs.calculate_module_scores’. The resulting scores were used to visualize module activity on a UMAP embedding and to assess their distribution across cell types and samples.

Over-representation analysis (ORA) of genes included in the Hotspot coexpression modules was performed using gseapy v1.1.8 through the gp.enrichr function. Gene set collections were obtained from Enrichr: Gene Ontology (GO) Biological_Process_2025 and Reactome_Pathways_2024. To ensure cross-species comparability, gene identifiers in these collections were mapped to mouse orthologs using the organism = “mouse” parameter. Enrichment significance was assessed using Fisher’s exact test with Benjamini–Hochberg correction for multiple testing. Terms with an adjusted *p*-value < 0.05 were considered significantly enriched.

### 2.7. Cellular Perturbation Analysis Using Augur

To quantify transcriptomic separability between species within each cell type, Augur, as implemented in pertpy v0.7.0 under Python 3.12, was applied. A Random Forest model was used as the classifier and trained on the top 2000 highly variable genes. The lymphatic endothelial cell (LEC) subtype population was excluded because of the insufficient number of cells. The area under the receiver operating characteristic curve (AUC) was calculated using the built-in cross-validation procedure. The resulting AUC values reflect the ability of the model to predict species identity (*A. cahirinus* versus *M. musculus*) from transcriptomic profiles within a given cell type.

### 2.8. Gene Set Enrichment Analysis

Gene set enrichment analysis was performed using blitzgsea (v.1.3.40). The GO_Biological_Process_2025 and Reactome_Pathways_2024 gene set libraries were retrieved through the Enrichr API implemented in gseapy (v.1.0.3). Enrichment was calculated from ranked gene lists for Gene Ontology Biological Process and Reactome Pathways terms. Terms with an adjusted *p*-value < 0.05 were considered significantly enriched.

### 2.9. Focused Re-Integration of Ventricular Cardiomyocytes

To resolve the intrapopulation heterogeneity of ventricular cardiomyocytes, this cell population was extracted from the integrated atlas based on cell type annotation and analyzed separately. For the cardiomyocyte subset, highly variable genes were re-selected using sc.pp.highly_variable_genes (n_top_genes = 2000), followed by principal component analysis using sc.tl.pca (n_comps = 50). Batch-effect correction was then performed with Harmony using sc.external.pp.harmony_integrate, with sample identifier used as the batch variable. A k-nearest neighbor graph was constructed using sc.pp.neighbors (n_neighbors = 15), followed by UMAP embedding with sc.tl.umap and Leiden clustering with sc.tl.leiden. The resulting clusters were used to analyze intrapopulation heterogeneity within ventricular cardiomyocytes.

## 3. Results

### 3.1. Interspecies Data Integration and Annotation of Cell Populations in the Intact Myocardium

**Figure 1:**
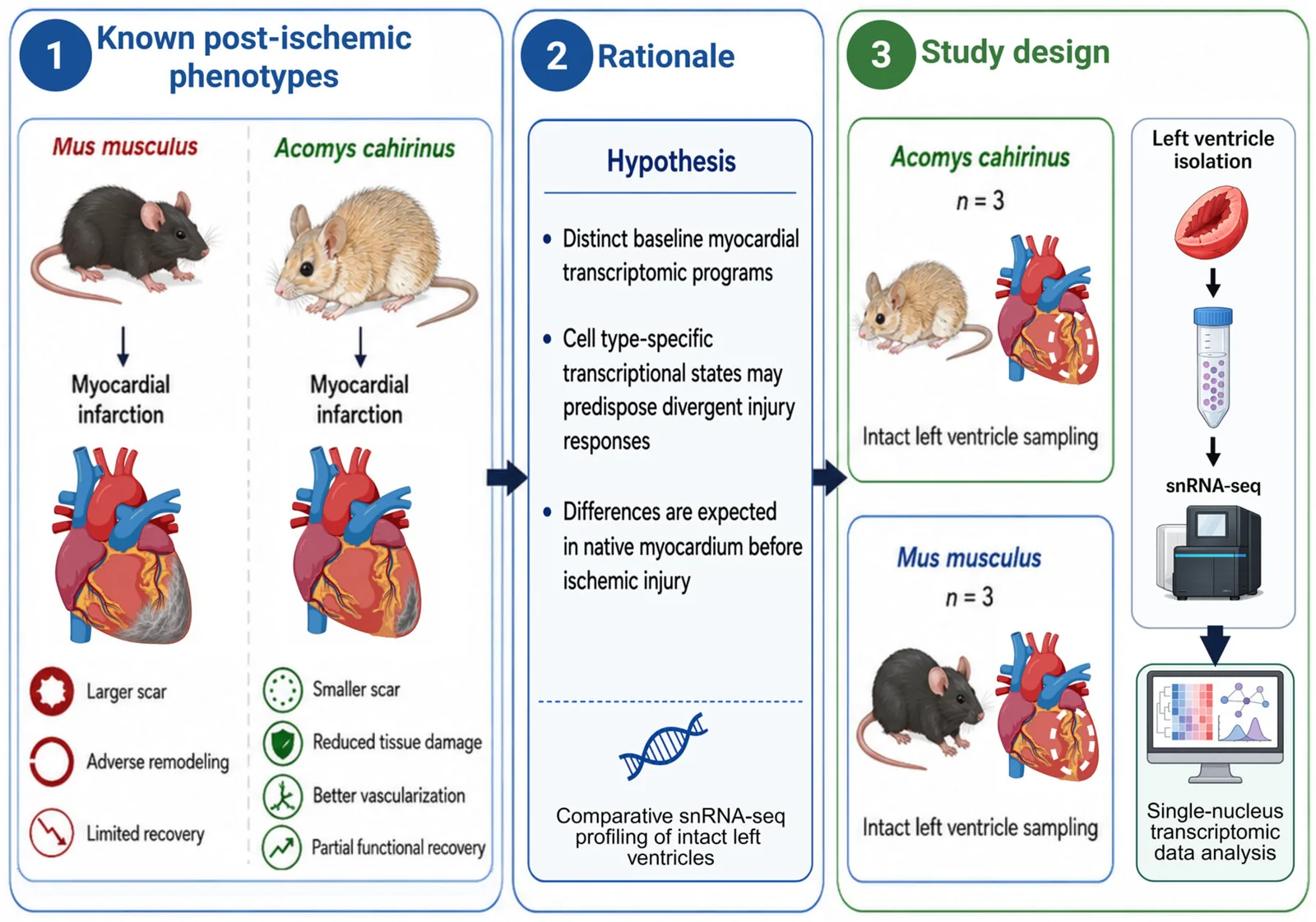
Overview of the experimental design. The scheme summarizes the post ischemic cardiac phenotypes that motivated the study, the sampling of intact left ventricles from adult *A. cahirinus cahirinus* and *Mus musculus*, nuclei isolation, single-nucleus RNA sequencing and subsequent analytical steps. These steps included quality control, data integration, cell type annotation, UMAP visualization, correlation analysis, assessment of interspecies separability using Augur, coexpression module detection and Gene Ontology enrichment analysis. RNA—Ribonucleic acid, UMAP—Uniform Manifold Approximation and Projection.

Comparative analysis of the intact left ventricle comprised an assessment of the overall cellular composition of the myocardium in both species, followed by evaluation of cell type-specific interspecies differences. Intact myocardial samples from *A. cahirinus* and *M. musculus* were analyzed by single-nucleus RNA sequencing, followed by data integration, annotation of cell populations, assessment of interspecies transcriptomic separation, and analysis of gene sets associated with specific cellular functions (Figure 1). Cardiomyocytes were predefined as the population of interest for subsequent cross-species comparison.

**Figure 2:**
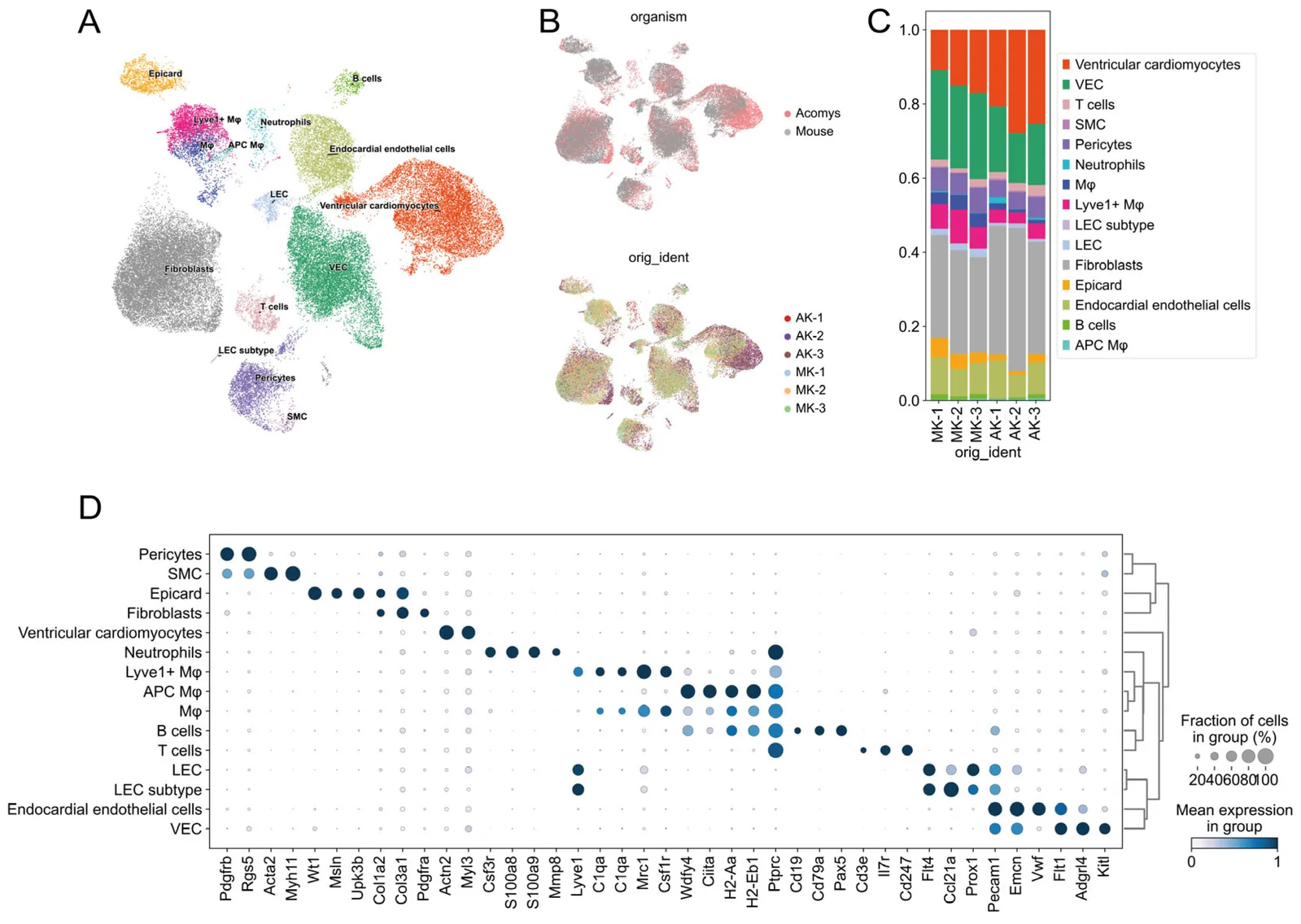
Interspecies integration of snRNA-seq data from the left ventricle of *A. cahirinus* and *M. musculus*, annotated with major cell populations. (**A**). UMAP projection of the integrated nuclear dataset annotated with the major myocardial cell populations. The identified populations included ventricular cardiomyocytes, fibroblasts, vascular, endocardial, and lymphatic endothelial cells, pericytes, smooth Muscle cells, epicardial cells, and immune populations, including macrophages, Lyve1^+^ macrophages, APC-like macrophages, neutrophils, T-lymphocytes, and B-lymphocytes. (**B**). Distribution of nuclei by species and individual sample on the shared UMAP projection. The upper panel shows the contribution of *A. cahirinus* and *M. musculus*, whereas the lower panel shows the distribution of individual biological replicates. (**C**). Proportions of annotated cell populations in each sample. Bars show the relative contribution of the major cell populations in *M. musculus* and *A. cahirinus* samples. (**D**). Expression of marker genes used to confirm cell-type annotations. Dot size represents the proportion of nuclei with detectable expression of the corresponding gene, and color intensity indicates the average expression level. APC—antigen-presenting cell, AK-1–AK-3—individual *A. cahirinus* samples, LEC—lymphatic endothelial cell, Mφ—macrophage, MK-1–MK-3—Individual *M. musculus* samples, SMC—smooth muscle cell, snRNA-seq—single-nucleus RNA sequencing, UMAP—Uniform Manifold Approximation and Projection, VEC—vascular endothelial cell.

The integrated atlas of the left ventricular nuclei of *A. cahirinus* and *M. musculus* was generated after quality control and the exclusion of clusters showing signs of duplicates or background RNA contamination (Figure 2). The UMAP projection resolved the major cell populations of the intact myocardium, including ventricular cardiomyocytes, fibroblasts, vascular, endocardial, and lymphatic endothelial cells, pericytes, smooth Muscle cells, epicardial cells, and immune cells (Figure 2A). These populations were detected in both species, and the distribution of nuclei by species and individual biological sample showed that the major clusters contained nuclei from both species and were not driven by a single sample (Figure 2B). Relative proportions of annotated cell populations across individual samples and expression patterns of established marker genes supported the consistency of cell type annotations (Figure 2C,D). The integration did not result in complete intermixing of nuclei from the two species, allowing the overall cellular organization of the myocardium to be retained while preserving the ability to assess interspecies transcriptomic differences.

### 3.2. Interspecies Separation of Cell Populations and Global Structure of Sample Transcriptomic Profiles

**Figure 3:**
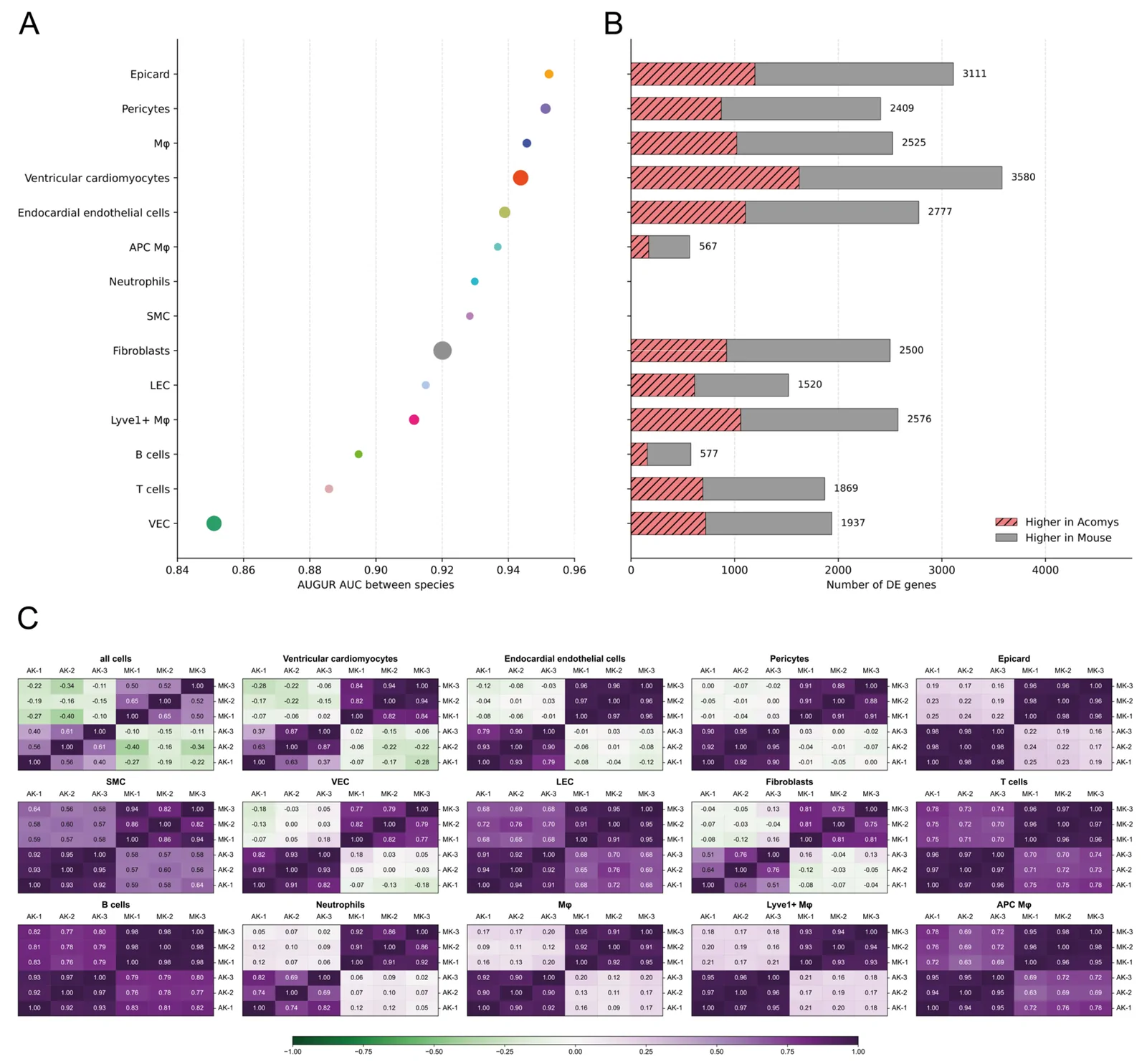
Cell type-specific interspecies differences in transcriptomic profiles of the intact myocardium in *A. cahirinus* and *M. musculus*. (**A**). Interspecies distinctiveness of transcriptomic profiles in individual cell populations, as measured by Augur AUC values. (**B**). Number of differentially expressed genes between *A. cahirinus* and *M. musculus* in individual cell populations. Hatched segments indicate genes with higher expression in *A. cahirinus*, whereas gray segments indicate genes with higher expression in *M. musculus*. Values are not shown for neutrophils or smooth muscle cells due to insufficient numbers of nuclei for reliable pseudobulk statistical analysis. (**C**). Correlation matrices of transcriptomic profiles across individual samples for the complete nuclear dataset and individual cell populations. Color represents the correlation coefficient between samples. AK-1–AK-3—individual *A. cahirinus* samples, APC—antigen-presenting cell, AUC—area under the receiver operating characteristic curve, DE—differentially expressed, LEC—lymphatic endothelial cell, Mφ—macrophage, MK-1–MK-3—individual *M. musculus* samples, SMC—smooth muscle cell, VEC—vascular endothelial cell.

To assess interspecies transcriptional differences within each cell type, classifier AUC values were calculated using the Augur module implemented in pertpy. AUC values remained high across all major cell populations (0.85 < AUC < 0.95), indicating consistent discrimination between the transcriptomic profiles of individual *A. cahirinus* and *M. musculus* nuclei within the same annotated cell types. The highest interspecies differences were observed in epicardial cells, pericytes, the macrophage compartment, and ventricular cardiomyocytes. Slightly lower, but still clearly elevated, AUC values were detected in T lymphocytes, B lymphocytes, and several vascular populations (Figure 3A).

The number of differentially expressed genes between *A. cahirinus* and *M. musculus* was also calculated for each cell population. Genes were included if the absolute log2 fold change (log2FC) was at least 1, the adjusted *p*-value was below 0.05, and expression was detected in at least 5% of nuclei in at least one of the two groups. Ventricular cardiomyocytes had the largest number of differentially expressed genes, with 3580 genes identified. A total of 3111 were found in epicardial cells, 2777 in endocardial endothelial cells, 2576 in Lyve1^+^ perivascular macrophages, 2525 in the macrophage compartment, 2500 in fibroblasts, and 2409 in pericytes. Vascular endothelial cells had 1937 differentially expressed genes, T lymphocytes 1869, and lymphatic endothelial cells 1520. The lowest numbers among the populations shown were detected in B lymphocytes and APC-like macrophages, with 577 and 567 genes, respectively (Figure 3B). Neutrophils and smooth muscle cells were excluded from differential expression analysis because their limited numbers of nuclei did not support reliable pseudobulk statistical analysis. In most cell compartments, genes with higher expression in *M. musculus* accounted for a substantial proportion of the differentially expressed genes, although genes with higher expression in *A. cahirinus* were also present across all major populations.

At the level of individual samples, interspecies separation was further assessed by correlation analysis of gene expression profiles. Correlation matrices were generated for the complete dataset and separately for each annotated cell population (Figure 3C). The matrices showed consistent within-species concordance among biological replicates and retention of the species-specific signal when cell compartments were analyzed separately. This correlation pattern indicated that the interspecies differences could not be attributed solely to variation in overall cellular composition but persisted within the transcriptomic profiles of individual cell types. Concordant results from the three analytical measures, including Augur AUC, the number of differentially expressed genes, and the correlation structure of individual samples, supported the cell type-specific nature of interspecies differences in the intact myocardium. Their magnitude varied among cell populations but remained evident despite the comparable overall composition of the integrated atlas.

### 3.3. Identification of Cell Type-Specific Coexpression Modules

**Figure 4:**
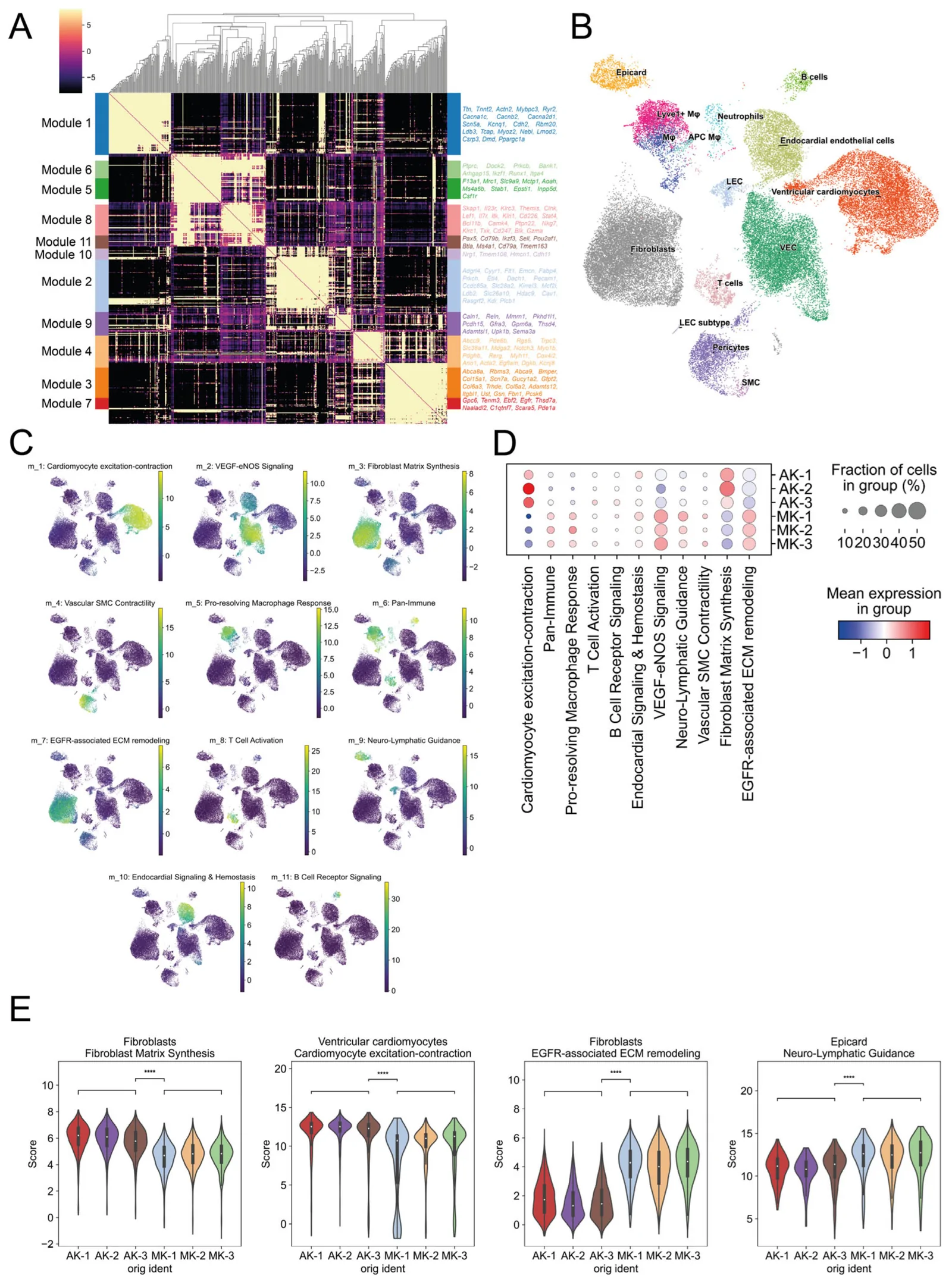
Coexpression modules associated with the major cell populations of the intact left ventricle in *A. cahirinus* and *M. musculus*. (**A**). Gene coexpression matrix with hierarchical clustering and identification of 11 coexpression modules. The key genes for each module are shown on the right. (**B**). UMAP projection of the integrated set of nuclei annotated with major myocardial cell populations. (**C**). Projection of coexpression module scores onto the UMAP projection of the integrated set of nuclei. The spatial distribution of the modules corresponds to their cellular localization. (**D**). Comparison of module scores across individual *A. cahirinus* and *M. musculus* samples. Dot size indicates the proportion of nuclei with a high score for the corresponding module, and color intensity indicates the mean module score in the group. (**E**). Distribution of selected module scores in the corresponding cell populations at the level of individual samples. Shown are the Fibroblast Matrix Synthesis module in fibroblasts, the Cardiomyocyte Excitation–Contraction module in ventricular cardiomyocytes, the EGFR-associated extracellular matrix (ECM) Remodeling module in fibroblasts, and the Neuro-Lymphatic Guidance module in epicardial cells. **** *p* < 0.0001. AK-1–AK-3—individual *A. cahirinus* samples, ECM—extracellular matrix, EGFR—epidermal growth factor receptor, eNOS—endothelial nitric oxide synthase, M1–M11—coexpression modules 1–11, MK-1–MK-3—individual *M. musculus* samples, SMC—smooth muscle cell, UMAP—Uniform Manifold Approximation and Projection, VEGF—vascular endothelial growth factor.

To characterize the major interspecies differences at the level of coordinated transcriptional programs rather than individual differentially expressed genes, we performed coexpression module analysis. Modules were identified from coordinated changes in gene expression within the integrated dataset. The block structure of the coexpression matrix indicated that the identified gene groups formed stable modules rather than reflecting random variation in individual marker genes. For each module, we assessed the distribution of module scores across annotated cell populations, the leading genes, and the results of functional enrichment analysis. Modules were interpreted based on their leading genes, the distribution of module signals across the UMAP projection, and ORA results. Each module was then assigned a descriptive functional name. Eleven coexpression modules were identified, each showing clear cell-type specificity and, in most cases, association with one or more related cell types (Figure 4A). Module M1 was associated with ventricular cardiomyocytes, M3 with fibroblasts, M4 with pericytes and smooth Muscle cells, M5 with Lyve1^+^ macrophages, M8 with T lymphocytes, and M11 with B lymphocytes, whereas M2, M9, and M10 were predominantly associated with distinct endothelial populations. Module M6 showed a broader immune profile, whereas M7 was associated with fibroblast and epicardial-related compartments. Functional enrichment of M7 included categories related to epidermal growth factor receptor/Erb-B receptor tyrosine kinase (EGFR/ERBB) signaling and regulation of tissue responses. These results showed that the identified modules covered the major cell populations of the intact myocardium and represented stable cell type-specific transcriptional programs. Projection of the modules onto the UMAP further showed that their distribution was consistent with the cell-type annotation of the integrated atlas (Figure 4B,C).

Based on their leading genes (Figure 4A) and cellular localization (Figure 4B,C), the modules were functionally annotated. M1 was defined as a cardiomyocyte excitation–contraction, M2 as an vascular endothelial growth factor/endothelial nitric oxide synthase (VEGF/eNOS) signaling, M3 as a fibroblast matrix synthesis, M4 as a vascular smooth muscle cell (SMC) contractility, M5 as a pro-resolving macrophage response, M6 as a pan-immune, M7 as an EGFR-associated ECM remodeling, M8 as a T-cell activation, M9 as a neuro-lymphatic guidance, M10 as an endocardial signaling and hemostasis, and M11 as a B-cell receptor signaling (Figure 4A). The concordance between the cell-type localization of the modules and their leading gene composition supported their use for subsequent cross-species comparison of cell type-specific transcriptional programs between *A. cahirinus* and *M. musculus*. Module annotation was used not as a substitute for cell-type annotation but as an additional analytical layer for assessing coordinated transcriptional programs within already defined cell populations.

After determining the cellular localization, the activity of the modules was assessed in *A. cahirinus* and *M. musculus* samples. At the level of individual biological samples, Ak1, Ak2, and Ak3 exhibited higher scores for M1 and M3, corresponding to the cardiomyocyte excitation-contraction and fibroblast matrix synthesis modules, respectively. In Mk1, Mk2, and Mk3, higher scores were predominantly observed for M7 and M9 (Figure 4D). Interspecies differences were also evident in the cell type-specific distributions of selected module scores (Figure 4E). M1 was selected for subsequent focused analysis based on its localization within the ventricular cardiomyocyte population, the observed interspecies difference in module activity, the high Augur AUC value for this population, and the largest number of differentially expressed genes among the annotated cell types.

### 3.4. Focused Reintegration of Ventricular Cardiomyocytes Reveals Interspecies Differences in Transcriptional States and Functional Signatures

**Figure 5:**
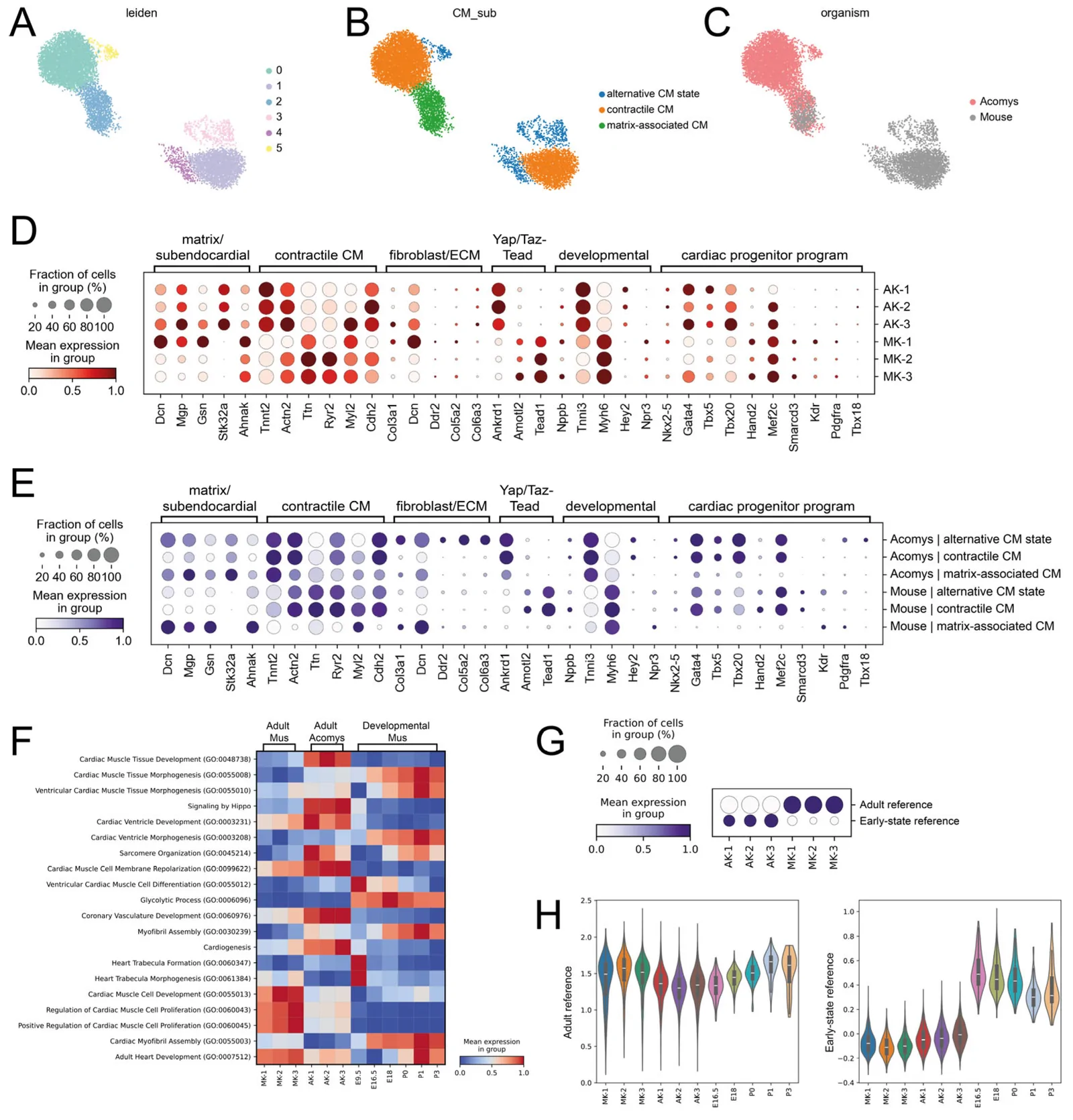
Reintegration and comparative analysis of ventricular cardiomyocytes in the intact left ventricle of *A. cahirinus* and *M. musculus*. (**A**). UMAP projection of the ventricular cardiomyocyte population after focused reintegration, with cluster annotations. (**B**). UMAP projection of nuclei after the clusters were combined into three transcriptional states comprising contractile CM, matrix-associated CM, and alternative CM state. (**C**). Distribution of *A. cahirinus* and *M. musculus* nuclei in the UMAP embedding of the reintegrated ventricular cardiomyocyte subset. (**D**). Representation of selected markers in individual samples of *A. cahirinus* and *M. musculus*. Genes associated with matrix/subendocardial traits, the cardiomyocyte contractile program, the fibroblast/ECM component, Yap/Taz-Tead-related regulation, developmental traits, and the cardiac progenitor-associated program are shown. The size of the dot reflects the proportion of nuclei with detectable gene expression, and the intensity of the staining reflects the average expression in the group. (**E**). Distribution of the markers shown in Panel (**D**) across the three annotated states of ventricular cardiomyocytes, separately for *A. cahirinus* and *M. musculus*. (**F**). Heatmap of selected Gene Ontology and Reactome categories in adult *M. musculus*, adult *A. cahirinus*, and publicly available developing *M. musculus* heart samples. The external developmental samples included embryonic and early postnatal stages E9.5, E16.5, E18, P0, P1, and P3. (**G**). Comparison of the Adult reference and Early-state reference signatures defined in this analysis across individual adult *A. cahirinus* and *M. musculus* samples. Dot size represents the proportion of nuclei within each group, and color intensity represents the mean signature score. (**H**). Distribution of the Adult reference and Early-state reference signatures defined in this analysis across *A. cahirinus*, adult *M. musculus*, and publicly available developmental *M. musculus* samples. Violin plots show the distribution of signature scores at the individual-nucleus level. AK-1–AK-3—individual *A. cahirinus* samples, CM—cardiomyocyte, ECM—extracellular matrix, E—embryonic day; GO—Gene Ontology, MK-1–MK-3—individual *M. musculus* samples, P—postnatal day, TAZ—transcriptional coactivator with PDZ-binding motif, TEAD—TEA domain transcription factor, UMAP—Uniform Manifold Approximation and Projection, YAP—Yes-associated protein.

Focused reintegration of ventricular cardiomyocytes was performed to clarify the internal structure of this population after it was isolated from the general integrated atlas. The UMAP projection identified six Leiden clusters (Figure 5A), which were grouped into three transcriptional states based on their marker profiles (Figure 5B). Most clusters were assigned to a state termed contractile (cardiomyocyte) CM, characterized by a cardiomyocyte contraction program. This assignment was supported by higher expression of *Tnnt2*, *Actn2*, *Ttn*, *Ryr2*, *Myl2*, and *Cdh2* together with other genes associated with the structural and contractile organization of cardiomyocytes (Figure 5D,E) [19,20]. Clusters characterized by higher expression of *Dcn*, *Mgp*, *Gsn*, *Stk32a*, and *Ahnak*, as well as selected fibroblast and ECM-associated transcripts, including *Ddr2*, *Col5a2*, and *Col6a3* [20,21,22,23,24], were assigned to the matrix-associated CM state (Figure 5D,E). The predominance of transcripts associated with fibroblasts, the extracellular matrix, and matrix remodeling supported interpretation of the matrix-associated CM state as a cardiomyocyte population transcriptionally linked to the matrix microenvironment rather than as a distinct fibroblast lineage. A further group of clusters, designated alternative CM state, differed from the predominant contractile state by higher expression of selected transcripts associated with regulation by yes-associated protein (YAP), transcriptional coactivator with PDZ-binding motif (TAZ), and TEA domain (TEAD) transcription factors (YAP/TAZ–TEAD) (Figure 5D,E), developmental programs, and cardiac progenitor-associated gene sets, including *Ankrd1*, *Amotl2*, *Tead1*, *Nppb*, *Tnni3*, *Myh6*, *Hey2*, *Npr3*, *Nkx2-5*, *Gata4*, *Tbx5*, *Tbx20*, *Hand2*, *Meis2*, and *Smarcd3* [20,25,26,27,28,29,30,31,32,33].

The distribution of nuclei by species revealed pronounced spatial separation between *A. cahirinus* and *M. musculus* in the UMAP embedding generated after focused reintegration of ventricular cardiomyocytes (Figure 5C). Spiny mouse nuclei were predominantly concentrated in the upper region of the embedding, whereas *M. musculus* nuclei occupied its lower region. Annotation of the transcriptional states further indicated that the interspecies differences involved not only separation of the two species in low-dimensional space but also differences in the representation of transcriptional programs within the ventricular cardiomyocyte population.

For functional comparison of cardiomyocyte profiles, a heatmap was generated using scores for selected Gene Ontology and Reactome categories related to cardiac muscle development and morphogenesis, Hippo signaling, ventricular development, sarcomere and myofibril organization, repolarization, glycolysis, vascular development, and regulation of cardiomyocyte proliferation. The analysis included adult *M. musculus* and *A. cahirinus* samples, together with publicly available data from developing *M. musculus* hearts at embryonic and early postnatal stages E9.5, E16.5, E18, P0, P1, and P3 reported by Feng et al. [34]. In the original study, mouse cardiac cells were profiled across consecutive embryonic and neonatal stages spanning E9.5 to P9. However, only selected representative stages were included to provide a more compact heatmap display. Adult samples from the two species and developing heart samples exhibited distinct profiles across the selected categories. For several terms related to cardiac Muscle development and morphogenesis, Hippo signaling, ventricular development, coronary vasculature, and cardiogenesis, the *A. cahirinus* profile showed partial concordance in the direction of change with the developing heart samples and differed from adult *M. musculus*. The developing heart samples nevertheless retained a distinct distribution of GO category scores, including higher values for selected terms related to trabecular morphogenesis, glycolysis, ventricular cardiomyocyte differentiation, and myofibril assembly (Figure 5F).

To summarize the comparative analysis, two additional reference signatures, designated Adult reference and Early-state reference, were derived from published gene sets characterizing mature [35,36,37,38,39,40,41,42] and early cardiomyocyte states [34,35,36,37,43,44,45,46,47,48,49,50,51,52]. Unlike individual GO and Reactome categories, this approach enabled comparison of the samples using integrated gene sets associated with a mature cardiomyocyte profile and an early development-associated state, respectively. Adult reference scores were higher in adult *M. musculus* samples than in *A. cahirinus*. Conversely, Early-state reference scores were higher in Ak1, Ak2, and Ak3 than in Mk1, Mk2, and Mk3 (Figure 5G).

Inclusion of publicly available embryonic and early postnatal *M. musculus* samples allowed the position of *A. cahirinus* relative to the developmental series to be defined more precisely. Adult reference scores increased progressively across later postnatal and adult *M. musculus* samples. Spiny mouse samples occupied an intermediate position and had lower scores than adult *M. musculus* samples. The highest Early-state reference scores were observed in embryonic and early postnatal *M. musculus* samples. *A. cahirinus* samples were positioned below this group and closer to adult *M. musculus* samples, while retaining higher scores than Mk1, Mk2, and Mk3 (Figure 5H).

## 4. Discussion

The results showed that the intact left ventricles of *A. cahirinus* and *M. musculus* contained comparable sets of the major myocardial cell populations, as reproduced in the integrated cross-species atlas. The similar composition of these populations indicates that interspecies differences in the intact myocardium are associated predominantly with their molecular states rather than with overall myocardial cellular composition. This approach reflects the use of single-cell and single-nucleus transcriptomics to characterize tissue cellular composition, cellular transcriptional states, and functional differences within annotated populations [8,20]. This finding is relevant to the interpretation of the regenerative phenotype of spiny mouse. Previous studies have described scar-free skin wound healing, ear pinna regeneration, and a more favorable response to ischemic myocardial injury in *A. cahirinus* than in *M. musculus* [9,16,17,53]. The coexistence of a similar cellular composition in the intact left ventricle with pronounced interspecies transcriptional differences warranted further examination of baseline cell type-specific myocardial states. These findings indicate that species-specific molecular differences are already present in the intact *A. cahirinus* heart before experimental injury and may contribute to the subsequent proregenerative response.

The broadly comparable representation of the major cell populations in the intact myocardium of spiny mice and *M. musculus* did not preclude pronounced interspecies differences within annotated cell types. The reliability of this observation was assessed by combining three complementary analyses, including cell-type ranking with Augur [54,55], quantification of differentially expressed genes, and evaluation of the correlation structure across individual samples. The degree of separability between the two species varied substantially across the annotated cell populations. This distribution of the species-associated signal is consistent with the view that interspecies transcriptomic variation may differ considerably among cell types [56,57]. The distribution of differential expression indicated that species-specific features of the intact myocardium were not confined to a single cellular compartment. The interspecies signal was detected across several annotated populations, supporting a multicomponent, cell type-specific pattern rather than an alteration restricted to a single lineage. Correlation analysis of individual samples further reduced the likelihood that the observed separation resulted solely from integration into a shared atlas or from between-sample variability. The identified differences can therefore be regarded as a trustworthy feature of the analyzed dataset that remains evident within the annotated cell populations.

Genes showing coordinated changes in expression were grouped into coexpression modules, enabling the analysis to move from individual genes to functionally related transcriptional programs [58,59]. This level of analysis complemented the Augur and differential expression results by showing that interspecies differences involved not only individual genes but also broader cell-associated programs linked to specific cellular clusters. Among the identified modules, M1, associated with cardiomyocyte excitation and contraction, was selected as the primary focus of subsequent analyses. Its cell-type localization was consistent with the Augur and differential expression results, which identified ventricular cardiomyocytes as one of the populations with the most pronounced interspecies differences. Subsequent analyses therefore focused on the baseline state of ventricular cardiomyocytes and on the functional programs that differed between *A. cahirinus* and *M. musculus* within this population. This focus was consistent with previous studies of the spiny mouse heart, which reported smaller cardiomyocytes, a higher proportion of mononuclear cardiomyocytes, reduced T-tubule density, and features of a less mature cellular state compared with the *M. musculus* [16,17,60]. The consistency of these findings supported a shift from a broad assessment of interspecies differences to a more detailed level of analysis.

**Figure 6:**
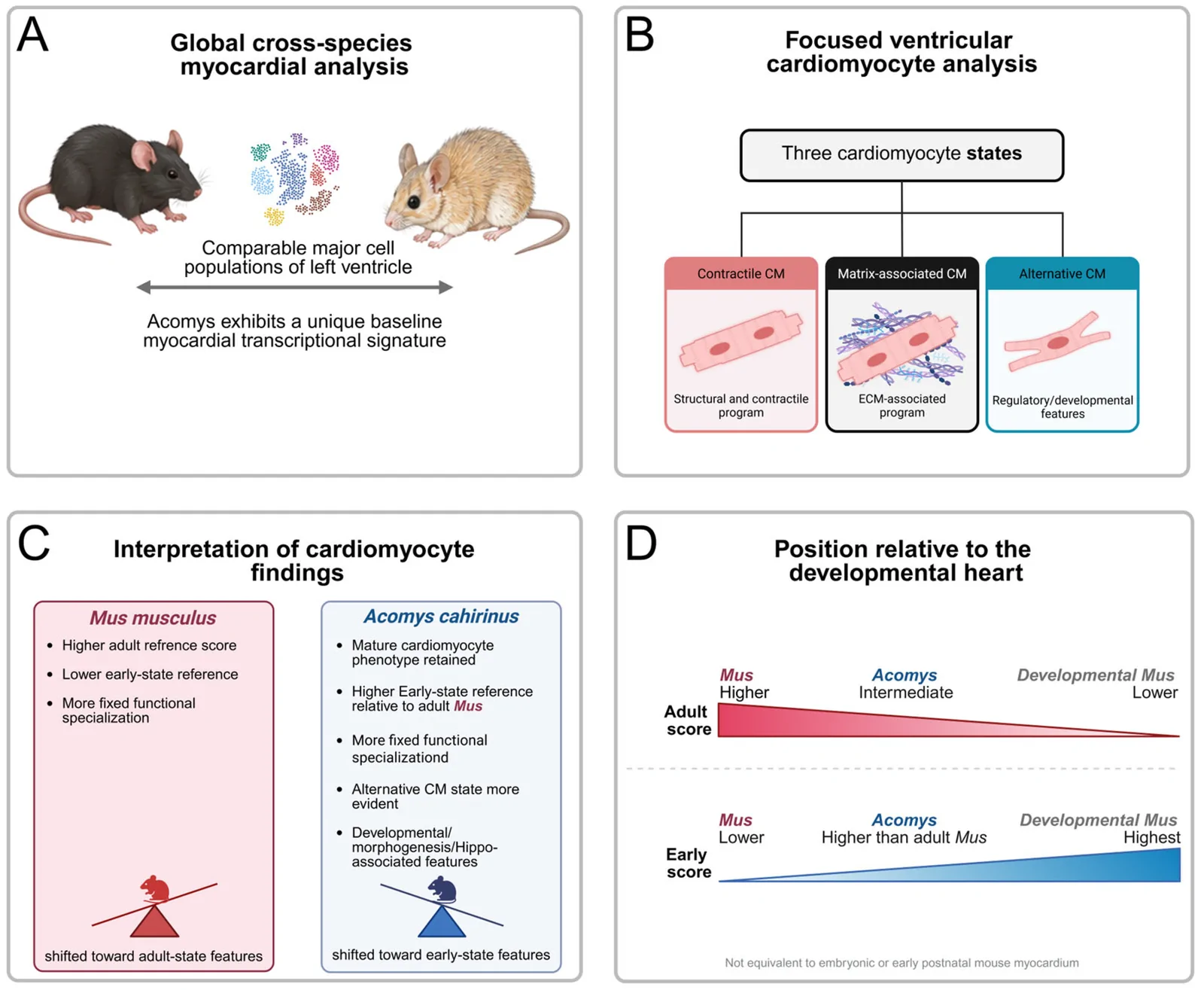
Summary of baseline interspecies differences in the intact left ventricle of *A. cahirinus* and *M. musculus*. (**A**). Global cross-species analysis showing comparable major myocardial cell populations and a distinct baseline transcriptional profile in *A. cahirinus*. (**B**). Three cardiomyocyte states identified by focused reintegration, including contractile CM, matrix-associated CM, and alternative CM state. (**C**). Summary of cardiomyocyte differences between species. Adult *A. cahirinus* retained a mature phenotype but differed from adult *M. musculus* in reference scores, functional specialization, and developmental, morphogenetic, and Hippo-associated features. (**D**). Relative positioning of adult *A. cahirinus*, adult *M. musculus*, and developing *M. musculus* according to Adult reference and Early-state reference scores. *A. cahirinus* differed from adult *M. musculus* without corresponding directly to embryonic or early postnatal mouse myocardium. CM—cardiomyocyte, ECM—extracellular matrix.

Reintegration of the ventricular cardiomyocyte subset clarified the nature of the interspecies differences identified in the integrated atlas. This analysis was not intended to identify additional cell types, but to characterize functional states within the previously annotated cardiomyocyte population (Figure 6B). In cardiac single-cell and single-nucleus atlases, reanalysis of annotated populations is commonly used to resolve variation associated with anatomical location, functional specialization, and cell regulatory programs [61]. The data did not support a binary interpretation in which *A. cahirinus* cardiomyocytes are classified as immature and *M. musculus* cardiomyocytes as mature. A more precise interpretation is that mature cardiomyocytes in the two species occupy distinct transcriptional states (Figure 6C). Spiny mouse cardiomyocytes retained features of mature cells, although a subset of nuclei diverged from the predominant contractile profile and exhibited regulatory features associated with developmental programs. Such an organization may reflect a less rigid determination of the program for mature functional specialization compared to *M. musculus*, but does not indicate a direct transition to an embryonic or progenitor state. Species-specific trajectories of cardiac maturation may also contribute to this pattern, given the differences between the two species in the timing of embryonic and early postnatal development. *A. cahirinus* is a precocial rodent with a longer gestation and extensive prenatal organ maturation, whereas a substantial proportion of organ maturation in *M. musculus* occurs after birth [62]. In comparative developmental studies, such differences are described as interspecies shifts in the timing and trajectories of organ maturation in mammals [63]. Accordingly, adult myocardial maturation may result in different molecular states in the two species. The adult *A. cahirinus* profile should therefore not be interpreted as arrest at an early developmental stage of *M. musculus*, but as a distinct outcome of completed cardiomyocyte maturation (Figure 6D). This state may represent cardiomyocyte maturity in *A. cahirinus*, although it corresponds less closely to the canonical adult *M. musculus* profile and retains selected development-associated features. This interpretation is consistent with the current view of cardiomyocyte maturity as a composite of structural, metabolic, electrophysiological, and regulatory features rather than a single linear measure [64].

To determine whether this deviation reflected greater similarity to earlier stages of cardiomyocyte development, the *A. cahirinus* profile was compared with publicly available developmental data from *M. musculus* [34]. Complete correspondence between embryonic or early postnatal mouse samples and the myocardium of adult *A. cahirinus* was not expected, given the differences in age, physiological state, and species. The relevant comparison therefore concerned concordant shifts in selected functional categories and reference signatures rather than direct equivalence of the profiles. The *A. cahirinus* profile showed partial convergence with developmental samples in categories related to morphogenesis, cardiogenesis, Hippo signaling, and ventricular development, but did not reproduce the complete embryonic or early postnatal pattern (Figure 5F). This distinction affects the interpretation of the reference signatures. The functional composition of both reference signatures was consistent with their intended biological rationale. Adult reference was predominantly associated with metabolic, contractile, and electrophysiological features of mature cardiomyocytes, whereas Early-state reference reflected programs related to the cell cycle, DNA replication, cardiogenesis, and YAP/TAZ-associated regulation (Figures S1 and S2). This correspondence justifies their use as comparative references but does not support equating either signature with a single canonical pathway or a fixed developmental stage. Lower Adult reference scores relative to adult *M. musculus* do not indicate loss of mature cardiomyocyte features, but rather reduced correspondence to the canonical adult *M. musculus* profile (Figure 5G). Early-state reference scores were higher in spiny mouse samples than in adult *M. musculus* samples, but remained below the range observed in embryonic and early postnatal developmental samples (Figure 5H). The *A. cahirinus* profile therefore cannot be regarded as a direct analogue of an embryonic or early postnatal state. Figure 5F–H indicates that *A. cahirinus* occupies a position distinct from adult *M. musculus*, although the observed differences cannot be reduced to a simple fetal-like or progenitor-like program.

The alternative CM state warrants separate consideration following the broader assessment of the reference programs. In the UMAP embedding of the reintegrated ventricular cardiomyocyte subset, this state formed a discrete region within the cardiomyocyte compartment. It retained some features of contractile CM but showed greater representation of regulatory, developmental, and YAP/TAZ–TEAD-associated features (Figure 5E). In regenerative models, myocardial repair may involve partial dedifferentiation of existing cardiomyocytes, reorganization of sarcomeric structure, and engagement of Hippo/YAP-dependent mechanisms [65,66,67]. The snRNA-seq data do not establish that dedifferentiation, proliferation, or activation of a regenerative program occurs in the intact *A. cahirinus* myocardium. The observed pattern should instead be interpreted as a baseline transcriptional state that may influence the subsequent cardiomyocyte response to injury. The interpretation of the matrix-associated CM state also requires specific qualification. The features underlying this annotation are predominantly associated with fibroblasts, the extracellular matrix, and matrix remodeling. This state should therefore not be regarded as a distinct fibroblast lineage within the cardiomyocyte population. In the present analysis, the annotation denotes a subset of cardiomyocyte nuclei with high expression of matrix-associated transcripts. The precise nature of this state requires further validation, because snRNA-seq data cannot unambiguously distinguish a genuine intermediate state from the influence of the local microenvironment or a technical background signal.

These data should be considered a feature of the pre-injury tissue state when evaluating the regenerative phenotype of the heart. Myocardial regeneration may involve a transient attenuation of mature cardiomyocyte specialization, remodeling of the contractile apparatus and an increased capacity for cardiomyocyte state transitions. In the Danio rerio heart regeneration model, new cardiomyocytes arise through proliferation of pre-existing cells, accompanied by partial dedifferentiation and sarcomere remodeling [65]. In mammalian experimental models, the Hippo signaling pathway and its effector Yap are involved in the regulation of cardiomyocyte proliferation, cardiac growth, and myocardial repair [66,67,68,69,70]. However, in the present study, the higher representation of the Hippo Signaling category in *A. cahirinus* does not allow us to determine the direction of activity of this pathway. It only indicates a difference in the transcriptional abundance of genes associated with Hippo signaling and requires verification at the level of nuclear localization of Yap and Taz, phosphorylation of pathway components, and the functional activity of cardiomyocytes. The combination of more pronounced categories associated with Hippo, development, and morphogenesis in *A. cahirinus*, with a different level of features of mature functional specialization, may indicate a more pliable initial state of ventricular cardiomyocytes. This interpretation is consistent with data indicating that the mechanical properties of the extracellular matrix, cell shape, Rho GTPase activity, and actomyosin cytoskeletal tension are involved in the regulation of Yap and Taz [71]. The proposed interpretation remains preliminary, as the current study did not evaluate the nuclear localization of Yap and Taz, the phosphorylation of Hippo pathway components, the mechanical properties of the tissue, or the ability of cardiomyocytes to re-enter cell cycle. Ventricular *A. cahirinus* cardiomyocytes should be considered as one possible component of a more favorable post-ischemic phenotype. Verifying this hypothesis requires functional, spatial, and protein-based methods.

The stromal and extracellular matrix compartment of the intact myocardium also warrants separate consideration. In an *A. cahirinus* model of myocardial infarction, the more favorable postischemic outcome [16] was previously associated not with the absence of scar formation, but with distinct organization of scar tissue and the extracellular matrix [72]. Following infarction, *A. cahirinus* showed less progression of the scarred area, preservation of left ventricular wall thickness, higher collagen content, increased collagen cross-linking, a greater proportion of mature fibers, and a more undulated matrix organization than *M. musculus* [17]. These data indicate that it is not simply the suppression of the matrix response that is important, but rather the composition, maturity, spatial organization, and mechanical properties of the scar matrix. Additional studies of this model showed that scars in *A. cahirinus* and *M. musculus* differed in proteomic composition and mechanical properties. The major fibrillar collagens Col1A1 and Col3A1 were increased in both species, whereas scar tissue in *M. musculus* was enriched in additional collagens and secreted profibrotic mediators. By contrast, *A. cahirinus* scars remained less stiff and retained a more undulated organization of matrix fibers. During transition to a myofibroblast state, *A. cahirinus* fibroblasts exhibited weaker induction of profibrotic genes and a reduced response to high substrate stiffness in vitro [18]. These observations are consistent with interspecies differences in matrix mechanics and fibroblast sensitivity to mechanical cues [73].

This concept is consistent with the overall role of the extracellular matrix in myocardial repair following ischemic injury. In the intact heart, the matrix provides mechanical support to the tissue, contributes to the organization of the cellular microenvironment, and mediates the transmission of regulatory signals [74]. The remodeling that occurs after a myocardial infarction affects the inflammatory response, leukocyte infiltration, fibroblast activation, the formation of a temporary matrix, angiogenesis, and the maturation of the collagen scar [74]. The functional consequences of the matrix response depend on collagen content and on the balance among collagen synthesis, degradation, cross-linking, maturation, and the spatial organization of collagen fibers [17,74]. Moreover, extracellular matrix genes constitute one of the major sources of heterogeneity among fibroblasts in single-cell transcriptomic datasets [23]. In the present dataset, the higher activity of M3 in spiny mouse indicates baseline differences in fibroblast and extracellular matrix programs in the intact left ventricle. Because M3 was associated with fibroblasts and extracellular matrix genes, the stromal compartment may differ molecularly from that of *M. musculus* before injury. These data do not demonstrate an antifibrotic or pro-regenerative state, but suggest that *A. cahirinus* fibroblasts may contribute, together with ventricular cardiomyocytes, to the more favorable postischemic phenotype. This possibility should be examined by spatial analysis of collagen organization, extracellular matrix proteomics, measurement of tissue mechanical properties, and longitudinal profiling of fibroblast states after injury.

## 5. Limitations

The study has several limitations that must be considered when obtaining results. The analysis was performed on a limited number of biological replicates and included only the intact left ventricle. Thus, the identified differences characterize the initial transcriptome organization of the native myocardium but do not allow for direct assessment of the dynamics of the cellular response after ischemic injury. The association of the detected cardiomyocyte, fibroblast, vascular, and immune programs with the more favorable post-infarction phenotype of *A. cahirinus* remains hypothetical and requires testing injury models with multiple time points. RNA sequencing of individual nuclei reflects the transcriptional state of cell populations but does not allow for direct assessment of protein content, post-translational modifications, intracellular localization of signaling effects, and functional activity of cells. This category, associated with Hippo, Yap, and Taz, development, morphogenesis, and contractile function, should be considered transcriptional signatures. Consequently, meaningful protein-level validation of the Hippo- and extracellular matrix-associated findings would require matched uninjured and infarcted hearts from both species collected at relevant post-injury time points. They do not demonstrate activation of the relevant signaling pathways, changes in the structure of intercellular contacts, or the ability of cardiomyocytes to resume the cell cycle.

Cross-species comparison of functional categories was based on *M. musculus* orthologs and the GO Biological Process and Reactome Pathways databases. This strategy enables comparison between species but may reduce sensitivity to species-specific genes, regulatory elements, and programs that are insufficiently represented in current annotations. Estimates of coexpression module activity and functional category scores also depend on genome annotation quality, integration parameters, selected thresholds, and the composition of the analyzed cell populations. Reintegration of the ventricular cardiomyocyte subset identified three transcriptional states that were considered in the interpretation but were not treated as discrete, homogeneous ventricular cardiomyocyte subpopulations. Their biological basis requires further validation, because contributions from boundary transcriptional signals, ambient RNA, incomplete separation from adjacent cellular compartments, and integration-related effects cannot be fully excluded.

Comparison with publicly available developmental heart datasets was used as an external reference rather than a direct comparison of ontogenetic stages. Embryonic and early postnatal *M. musculus* samples differed from mature *A. cahirinus* samples in age, physiological state, data acquisition platform, and species origin. Given these limitations, similarities or differences in profiles in this analysis should be interpreted as relative shifts across functional categories and reference signatures, rather than as assignment of spiny mice to a specific stage of *M. musculus* development. The Adult Reference and Early-State Reference signatures represented working gene sets generated for this analysis. Their values depended on their composition, the quality of ortholog matching, and normalization parameters.

The interpretation of the stromal matrix component as a possible contributor to the more favorable post-ischemic phenotype of *A. cahirinus* also remains preliminary. In this study, this aspect was discussed as a hypothesis related to differences in the initial state of fibroblasts and matrix programs. The spatial organization of collagen, the composition of extracellular matrix proteins, the mechanical properties of the tissue, and the dynamics of fibroblast states after injury were not assessed and require separate analysis.

## 6. Conclusions

Conceptually, the obtained results clarify our understanding of the regenerative phenotype of *A. cahirinus*. Previously, most studies focused on the cardiac response to injury, while the baseline organization of the native myocardium remained less well-characterized. The presented data demonstrate that interspecies differences are already evident in the native heart and simultaneously affect several key cell populations. Thus, the more favorable response of spiny mice to myocardial injury may be associated not only with the programs activated after injury but also with the initial cell-specific organization of the myocardium. This interpretation requires experimental verification but is consistent with the concept of *A. cahirinus* as a model for regeneration in adult mammals and with published data on the heart of this species.

## Data Availability

The processed single-nucleus RNA-seq data reported in this study have been deposited in the OMIX, China National Center for Bioinformation/Beijing Institute of Genomics, Chinese Academy of Sciences (https://ngdc.cncb.ac.cn/omix; accession no. OMIX020039).

## Supplementary Materials

The following supporting information can be downloaded at https://www.mdpi.com/article/10.3390/genes17091071/s1, Figure S1: Over-representation analysis of the Early-state reference cardiomyocyte gene set; Figure S2: Over-representation analysis of the Adult reference cardiomyocyte gene set.
