# Supplementary material for: Comparative Single-Nucleus Transcriptomic Analysis of the Intact Left Ventricle in Acomys cahirinus and Mus musculus
Source: Genes (Basel). 2026 Sep 4;17(9):1071. doi: 10.3390/genes17091071 (PMC13606643; doi:10.3390/genes17091071)
Supplement: Supplementary file 1 [file genes-17-01071-s001.zip › Figure S1.pdf]

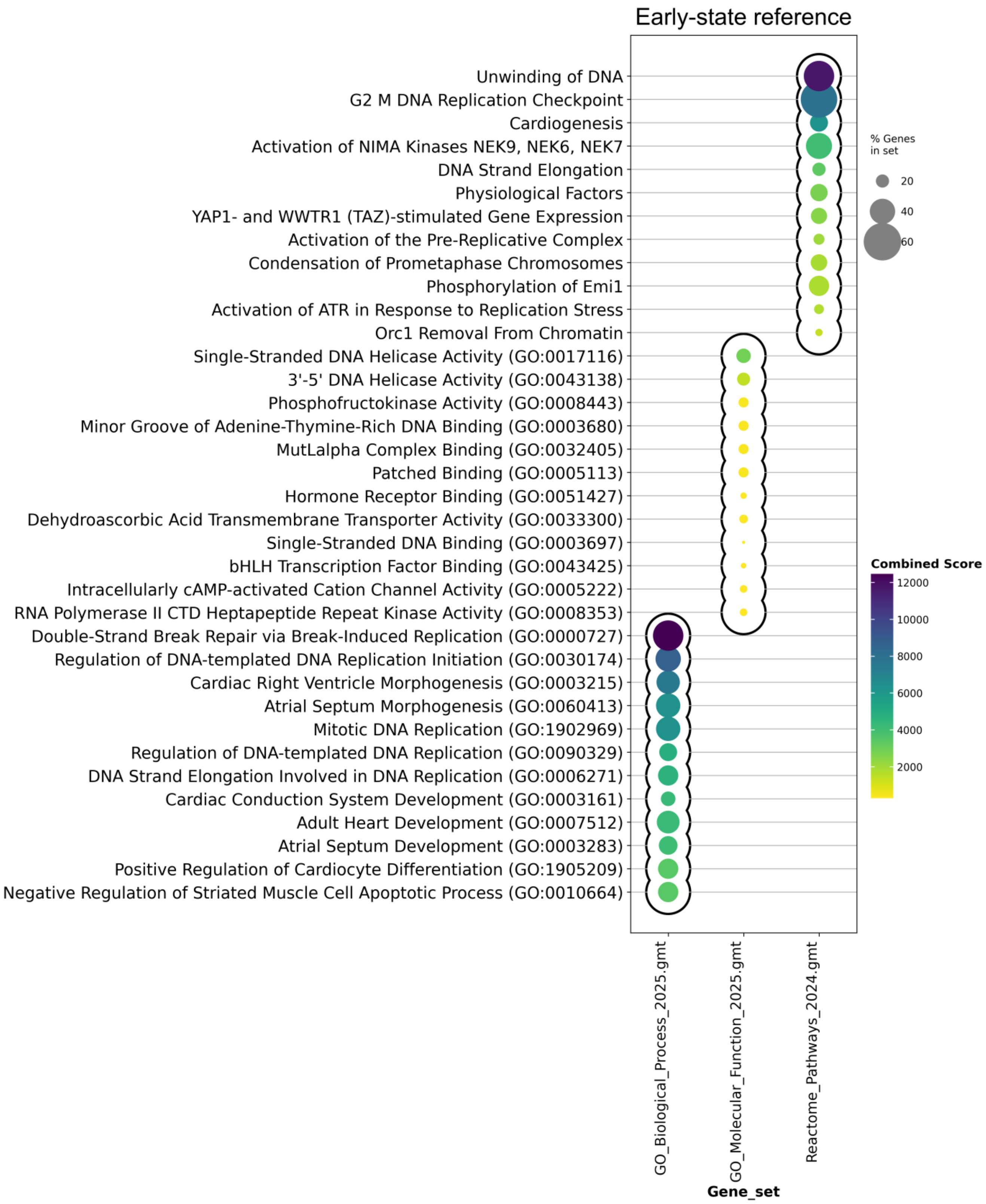

Figure S1. Over-representation analysis of the Early-state reference cardiomyocyte gene set.
